# Comparison of GLIM, SGA, PG-SGA, and PNI in diagnosing malnutrition among hepatobiliary-pancreatic surgery patients

**DOI:** 10.3389/fnut.2023.1116243

**Published:** 2023-01-24

**Authors:** Lingmei Zhou, Jianying Fu, Zhen Ding, Kemei Jin, Runjingxing Wu, Ling Xiao Ye

**Affiliations:** ^1^Clincal Nutrition Department, Ningbo Medical Center Li Huili Hospital, Ningbo, Zhejiang, China; ^2^Department of Hepatobiliary-Pancreatic Surgery, Ningbo Medical Center Li Huili Hospital, Ningbo, Zhejiang, China; ^3^Nursing Department, Ningbo Medical Center Li Huili Hospital, Ningbo, Zhejiang, China

**Keywords:** GLIM, PG-SGA, SGA, PNI, malnutrition, hepatobiliary-pancreatic surgery, hospitalized patients

## Abstract

**Objective:**

To compare the diagnostic value of four tools—the Global Leadership Initiative on Malnutrition (GLIM) criteria, the subjective global assessment (SGA), patient-generated subjective global assessment (PG-SGA), and prognostic nutritional index (PNI) in malnutrition among hospitalized patients undergoing hepatobiliary-pancreatic surgery. Meanwhile, to observe the nutritional intervention of these patients.

**Methods:**

Present study was a cross-sectional study, including 506 hospitalized patients who underwent hepatobiliary-pancreatic surgery between December 2020 and February 2022 at Ningbo Medical Center Lihuili Hospital, China. The incidence rate of malnutrition was diagnosed using the four tools. The consistency of the four tools was analyzed by Cohen's kappa statistic. Data, including nutritional characteristics and nutritional interventions, were collected. The nutritional intervention was observed according to the principles of Five Steps Nutritional Treatment.

**Results:**

The prevalence was 36.75, 44.58, and 60.24%, as diagnosed by the GLIM, PG-SGA, and PNI, respectively, among 332 tumor patients. Among the 174 non-tumor patients, the prevalence was 9.77, 10.92, and 32.18% as diagnosed by the GLIM, SGA, and PNI. The diagnostic concordance of PG-SGA and GLIM was higher (Kappa = 0.814, <0.001) than SGA vs. GLIM (Kappa = 0.752, *P* < 0.001) and PNI vs. GLIM (Kappa = 0.265, *P* < 0.001). The univariate analysis revealed that older age, lower BMI and tumorous were significantly associated with nutritional risks and malnutrition. Among 170 patients with nutritional risk, most of patients (118/170, 69.41%) did not meet the nutritional support standard.

**Conclusion:**

The incidence of nutritional risk and malnutrition is high among patients with hepatobiliary and pancreatic diseases, specifically those with tumors. The GLIM showed the lowest prevalence of malnutrition among the four tools. The PG-SGA and GLIM had a relative high level of agreement. There was a low proportion of nutritional support in patients. More prospective and well-designed cohort studies are needed to confirm the relevance of these criteria in clinical practice in the future.

## 1. Introduction

Malnutrition has been diagnosed as a disease in the complication or comorbidity (CC)/major complication or comorbidity (MCC) catalog of China Healthcare Security Diagnosis Related Groups (CHS-DRGS) with disease codes E40-E46 (1). Nutritional intervention was an important part of multidisciplinary treatment to improve the prognosis of patients, including a shorter hospital stay, less post-operative complications, lower mortality and reduced medical costs (2–4). There were several tools available for the assessment of malnutrition, such as the subjective global assessment (SGA), patient-generated subjective global assessment (PG-SGA), and prognostic nutritional index (PNI). However, the diagnostic criteria have not been harmonized in recent years. In September 2018, the Global Leadership Initiative on Malnutrition (GLIM) was issued to reach a global consensus on the clinical diagnosis of malnutrition (5). It made a progress that the nutritional assessment developed from the subjective methods such as SGA and PG-SGA to diagnosis malnutrition by phenotypic and etiological indicators.

Hospitalized patients undergoing hepatobiliary-pancreatic surgery have a high risk of malnutrition (6). Complex changes in nutrient metabolism and varying degrees of malnutrition may occur in hepatobiliary and pancreatic diseases. Nutritional status in turn affects the occurrence, development and prognosis of the diseases to form a vicious circle. Therefore, it was vital to early diagnosis malnutrition and timely implement nutritional intervention in the multidisciplinary. The applicability and effectiveness of the GLIM criteria have been verified among hospitalized patients with stroke (7), chronic pulmonary obstructive diseases (COPD) (8), gastrointestinal surgery (9), and heart failure (10). However, malnutrition prevalence, as determined by GLIM, among hospitalized patients undergoing hepatobiliary-pancreatic surgery remains largely unknown. Therefore, we aimed to (1) assess its prevalence, as defined by the GLIM, among hospitalized patients undergoing hepatobiliary-pancreatic surgery; (2) compare the PG-SGA, SGA, and PNI with the GLIM criteria in diagnosing malnutrition among these patients; (3) observe the nutritional intervention in hospitalized patients; (4) analyze the factors influencing nutritional risk and malnutrition among these hospitalized patients. In summary, we hope that our research can provide a reference for the more accurate nutritional diagnosis and treatment management of this group.

## 2. Methods

### 2.1. Study design and participants

Present study was a single-center, cross-sectional study. It included hospitalized patients who underwent hepatobiliary surgery between December 2020 and February 2022 at Ningbo Medical Center Lihuili Hospital, China. patients included in this study must meet these criteria as follow: (1) aged 18 to 90 years; (2) hospitalized for more than 5 days; (3) conscious; (4) cooperate with nutritional assessment; The exclusion criteria included: (1) length of stay (LOS) of <5 days; (2) refusal to cooperate; and (3) incomplete data required by the study. The study was approved by the ethics committee of the Ningbo Medical Center Lihuili Hospital.

Finally, a total of 506 patients were finally enrolled, including 335 males (66.21%) and 171 females (33.79%). The mean age was 59.73 ± 12.46 years.

### 2.2. Data acquisition

Malnutrition risk was screened by a trained nutritionist using the Nutritional Risk Screening 2002 (NRS2002) score according to the ESPEN and CSPEN guidelines within the first day after admission (11). NRS2002 score was consisted of three parts including the nutritional status, the severity of illness and the age assessment. The total score of NRS2002 ranges from 0 to 7. NRS2002 score ≥3 indicated that the patient was nutritionally at-risk. Malnutrition was further defined using the GLIM, PNI, PG-SGA, and SGA criteria among participants with nutritional risk.

Clinical data including disease diagnosis, age, sex, height, body weight, body mass index (BMI), length of stay (LOS) and other regular laboratory data were collected. BMI is an evaluation index of overweight and obesity, while the cut-off points defining overweight and obesity are different for Eastern Asian and Western populations. Because our study participants were all Chinese individuals,BMI was categorized as underweight (<18.5 kg/m^2^), normal weight (18.5 kg/m^2^ ≤ BMI <24.0 kg/m^2^), overweight (24.0 kg/m^2^ ≤ BMI <28.0 kg/m^2^) and obese (BMI ≥ 28.0 kg/m^2^) according to the Chinese Working Group on Obesity (WGOC) categories (12). Laboratory test results included total protein, albumin, hemoglobin, leukocyte count, triglycerides (TG), and total cholesterol (TC).

### 2.3. Diagnostic tools for malnutrition (GLIM, PG-SGA, SGA, and PNI)

The GLIM assessment criteria includes two parts:

- Phenotypic criteria (at least one of three characteristics):

① Unintentional weight loss: >5% within past 6 months or >10% beyond 6 months;

② Low body mass index (kg/m^2^): <20 if <70 years, or <22 if >70 years; Asia: <18.5 if <70 years, or <20 if >70 years;

③ Reduced muscle mass: reduced by validated body composition measuring techniques.

However, we excluded measurements of total body muscle mass because there was no cut-off point based on clinical studies for the fat-free mass index (FFMI) in China (13).

- Etiologic (presence of at least one criterion):

① Reduced food intake or assimilation: ≤ 50% of ER >1 week, or any reduction for >2 weeks, or any chronic GI condition that adversely impacts food assimilation or absorption;
② Inflammation: Acute disease/injury or chronic disease-related.

The PG-SGA includes seven boxes. Boxes 1–4 are designed to be completed by the patient. Boxes 5–7 are completed by the medical staff. The final score was obtained by adding each individual items. Among tumor patients in our study, a total score ≥4 was defined as malnutrition (14).

Subjective global assessment (SGA) is commonly used as a reliable and valid nutritional assessment tool to diagnose malnutrition (15, 16). It is a subjective assessment based on medical history and physical examination. The grading scale is divided into 3 levels, level A defines as well-nourished, level B defines as moderately malnourished, level C defines as severely malnourished. SGA was assessed among patients with non-tumor diseases.

The computational formula of PNI was: serum albumin (ALB) (g/L) + 5 ^*^ total lymphocyte count (10^9^/L), which was firstly proposed by Onodera et al. in 1984 (17). It was a comprehensive nutritional inflammatory indicator which can predict the prognostic of various solid tumors and cardiovascular diseases (18, 19), even the COVID-19 (20). According to the standard formulated by Onodera et al. a PNI <45 was considered malnutrition.

### 2.4. Concordance between the GLIM, SGA, PG-SGA, and PNI

We calculated the sensitivity, specificity, accuracy, and Cohen kappa coefficient (Kappa) to assess the diagnostic agreement. The value of kappa coefficient >0.8 defines as very good level of agreement, between 0.61 and 0.8 defines as good level of agreement, between 0.41 and 0.6 defines as moderate diagnostic agreement, between 0.21 and 0.4 defines as fair diagnostic agreement, and <0.2 defines as poor diagnostic agreement.

### 2.5. Statistical analysis

Continuous variables were expressed as mean ± standard deviation. The difference between groups were measured by *t*-test. Categorical variables were expressed as numbers (percentages) and difference between groups were measured by chi-square test. All statistical analyses were performed using SPSS 25.0, A *P* < 0.05 was treated as statistically significant.

## 3. Results

### 3.1. Study characteristics

According to the first diagnosis at admission, there were 10 types of diseases in hepatobiliary and pancreatic surgery section in our study, with the highest number of cases accounting for 43.68% (221/506) of liver cancer. The baseline characteristics of the study population are presented in Table 1.

**Table 1:** Baseline characteristics of the study population.

**Disease**	**N**	**Gender (male/female)**	**Age (years, $\bar{x}$ ±s)**
Liver cancer	221	181/40	60.40 ± 10.70
Pancreatic cancer	61	34/27	63.05 ± 11.77
Cholangiocarcinoma	34	21/13	64.65 ± 9.44
Gallbladder carcinoma	16	9/7	67.94 ± 8.39
Liver cirrhosis	14	12/2	58.00 ± 10.12
Post-orthotopic liver transplantation	11	8/3	54.73 ± 9.67
Cholecystitis	31	13/18	53.07 ± 15.02
Pancreatitis	4	1/3	53.25 ± 20.76
Cholangiolithiasis	30	15/15	63.71 ± 14.14
Cholecystolithiasis	84	41/43	54.42 ± 14.55
Total	506	335/171	59.73 ± 12.46

### 3.2. Prevalence of nutritional risk and malnutrition diagnosed by the four methods

Among the 506 participants, 170 patients with nutritional risk were diagnosed by the NRS2002, the nutritional risk incidence rate of 33.60% at admission. Patients with pancreatic cancer had the highest proportion of nutritional risks (65.57%), and that of nutritional risks among the 31 patients with cholecystitis was 0.00% (Table 2).

**Table 2:** Prevalence of nutritional risk in hepatobiliary-pancreatic surgery.

**Disease**	***N***	**Nutritional risk (NRS2002 ≥3)**	**(%)**
Liver cancer	221	79	35.75
Pancreatic cancer	61	40	65.57
Cholangiocarcinoma	34	19	55.88
Gallbladder carcinoma	16	9	56.25
Liver cirrhosis	14	7	50.00
Post-orthotopic liver transplantation	11	6	54.55
Cholecystitis	31	0	0.00
Pancreatitis	4	2	50.00
Cholangiolithiasis	30	3	10.00
Cholecystolithiasis	84	5	5.95
Total	506	170	33.60

Table 3 shows the prevalence of malnutrition diagnosed by GLIM, PNI, PG-SGA, and SGA. Among 332 tumor patients, malnutrition was diagnosed in 122 (36.75%), 200 (60.24%), and 148(44.58%) patients using the GLIM, PNI, and PG-SGA, respectively. Among 174 non-tumor patients, it was diagnosed in 17 (9.77%), 56 (32.18%), and 19 (10.92%) using the GLIM, PNI, and SGA, respectively.

**Table 3:** Prevalence of malnutrition in hepatobiliary-pancreatic surgery diagnosed by four methods.

**Disease**		***N***	***N*** **(%)**			
			**GLIM**	**PNI**	**PG-SGA**	**SGA**
Tumorous	Liver cancer	221	65(29.41%)	142 (64.25%)	79(35.75%)	**/**
	Pancreatic cancer	61	34(55.74%)	26(42.62%)	41(67.21%)	**/**
	Cholangiocarcinoma	34	16(47.06%)	25(73.53%)	19(55.88%)	**/**
	Gallbladder carcinoma	16	7(43.75%)	7(43.75%)	9 (56.25%)	**/**
	Total	332	122 (36.75%)	200(60.24%)	148 (44.58%)	**/**
Non-tumorous	Liver cirrhosis	14	7 (50%)	11(78.57%)	/	7 (50%)
	Post-orthotopic liver transplantation	11	4 (36.36%)	8(72.73%)	/	6 (54.55%)
	Cholecystitis	31	0 (0.00%)	5(16.13%)	/	0 (0.00%)
	Pancreatitis	4	0 (0.00%)	3(75.00%)	/	0 (0.00%)
	Cholangiolithiasis	30	3 (10.00%)	16(53.33%)	/	3 (10.00%)
	Cholecystolithiasis	84	3(3.57%)	13(15.48%)	/	3(3.57%)
	Total	174	17 (9.77%)	56(32.18%)	/	19 (10.92%)

### 3.3. The observation of nutritional interventions in hepatobiliary-pancreatic surgery

We also evaluated nutritional interventions in hepatobiliary-pancreatic surgery. Among 170 patients with nutritional risk, 26 received oral nutritional supplements (ONS), five received total enteral nutrition support (TEN), two received partial enteral and parenteral nutrition support (PEN + PPN), 16 patients received total parenteral nutrition support (TPN). Most (118/170, 69.41%) did not receive standard nutritional support (Table 4). Of the only 52 cases which received nutritional support, 46 were the patients with tumorous including 16 liver cancer patients, 17 pancreatic cancer patients, 9 cholangiocarcinoma patients and 4 gallbladder carcinoma patients. The other 6 patients without tumorous included 1 liver cirrhosis patient, 3 post-orthotopic liver transplantation patients, 1 cholangiolithiasis patient, and 1 cholecystolithiasis patient. There were 33% patients (17/52) who received the nutritional support during pre-surgery hospitalization and 67% patients (35/52) who received the nutritional support after surgery. We observed that clinical doctors were insufficiently aware of nutritional support and they did not follow the principles of nutritional interventions strictly. The bedside physicians mainly focused on the body weight and the serum albumin level instead of timely nutrition screening and assessment.

**Table 4:** The observation of nutritional interventions in hepatobiliary-pancreatic surgery.

**Disease**	**Nutritional risk (NRS2002 ≥3)**	**No nutritional support**	**ONS**	**TEN**	**PEN + PPN**	**TPN**
Liver cancer	79	63 (79.75%)	10 (12.66%)	2 (2.53%)	2 (2.53%)	2 (2.53%)
Pancreatic cancer	40	23 (57.50%)	5 (12.50%)	1 (2.50%)	1 (2.50%)	10 (25.00%)
Cholangiocarcinoma	19	10 (52.63%)	5 (26.32%)	0 (0.00%)	1 (5.26%)	3 (15.79%)
Gallbladder carcinoma	9	5 (55.56%)	3 (33.33%)	1 (11.11%)	0 (0.00%)	0 (0.00%)
Liver cirrhosis	7	6 (85.71%)	0 (0.00%)	0 (0.00%)	0 (0.00%)	1 (14.29%)
Post-orthotopic liver transplantation	6	3 (50%)	1 (16.67%)	1 (16.67%)	1 (16.67%)	0 (0.00%)
Cholecystitis	0	0 (0.00%)	0 (0.00%)	0 (0.00%)	0 (0.00%)	0 (0.00%)
Pancreatitis	2	2 (100%)	0 (0.00%)	0 (0.00%)	0 (0.00%)	0 (0.00%)
Cholangiolithiasis	3	2 (66.67%)	1 (33.33%)	0 (0.00%)	0 (0.00%)	0 (0.00%)
Cholecystolithiasis	5	4 (80.00%)	1 (20.00%)	0 (0.00%)	0 (0.00%)	0 (0.00%)
Total	170	118 (69.41%)	26 (15.29%)	5 (2.94%)	5 (2.94%)	16 (9.41%)

### 3.4. The relevance between the nutrition related indicators and the four methods

The data in Table 5 showed the relevance between the nutrition related indicators and the four methods. Of all the indicators, the level of serum albumin and hemoglobin in malnutrition group patients diagnosed by all the four tools were significantly lower than well-nourished group patients. The hs-CRP, an inflammation related indicator, was significantly higher in malnutrition group diagnosed by the GLIM, PG-SGA and PNI.

**Table 5:** Nutritional characteristics of the study population stratified by the GLIM, PNI, PG-SGA, and SGA.

**Characte-** **ristics**	**GLIM**				**PNI**				**PG-SGA**				**SGA**			
	**Mal** **nourished (*****n*** = **139)**	**well-** **nourished (*****n*** = **367)**	***t***	***P***	**Mal** **nourished (*****n*** = **256)**	**Well-** **nourished (*****n*** = **250)**	***t***	***P***	**Mal** **nourished (*****n*** = **148)**	**Well-** **nourished (*****n*** = **184)**	***t***	***P***	**Mal** **nourished (*****n*** = **19)**	**Well-** **nourished (*****n*** = **155)**	***t***	***P***
Age	63.81 ± 11.80	58.19 ± 12.39	4.62	0.000	61.77 ± 11.59	57.46 ± 13.15	3.90	0.000	63.74 ± 11.26	59.97 ± 10.22	3.19	0.002	62.58 ± 14.98	55.26 ± 14.22	2.10	0.037
LOS	11.53 ± 10.99	8.62 ± 7.20	2.89	0.004	10.92 ± 10.33	7.80 ± 5.55	4.10	0.000	10.53 ± 10.75	9.08 ± 7.27	1.46	0.145	16.16 ± 11.11	7.77 ± 6.08	3.23	0.000
BMI	20.41 ± 3.36	23.66 ± 3.31	9.83	0.000	22.13 ± 3.74	23.43 ± 3.38	4.10	0.000	20.87 ± 3.70	23.70 ± 3.03	7.49	0.000	20.86 ± 6.38	23.70 ± 2.91	1.92	0.070
Albumin	33.42 ± 5.93	38.53 ± 7.25	7.42	0.000	31.84 ± 5.04	42.54 ± 4.17	26.06	0.000	33.88 ± 6.32	37.82 ± 6.59	5.52	0.000	33.35 ± 6.42	40.62 ± 5.70	5.18	0.000
Hemoglobin	111.26 ± 23.01	126.00 ± 24.12	6.21	0.000	111.87 ± 21.54	134.01 ± 18.32	12.46	0.000	113.05 ± 23.45	125.60 ± 21.30	5.10	0.000	100.79 ± 18.96	131.04 ± 19.63	6.36	0.000
Triglyceride	1.14 ± 0.70	1.24 ± 0.97	1.14	0.254	1.18 ± 1.12	1.25 ± 0.61	0.85	0.396	1.09 ± 0.63	1.20 ± 0.75	1.46	0.146	1.03 ± 0.44	1.36 ± 1.25	1.12	0.264
Cholesterol	3.74 ± 1.41	4.15 ± 1.31	3.06	0.002	3.74 ± 1.57	4.35 ± 0.99	5.18	0.000	3.79 ± 1.39	4.06 ± 1.47	1.75	0.082	3.70 ± 1.42	4.29 ± 1.09	2.15	0.033
hs-CRP	35.86 ± 44.56	20.29 ± 36.94	3.67	0.000	34.18 ± 43.57	14.21 ± 31.94	5.87	0.000	35.25 ± 43.66	17.05 ± 28.85	4.36	0.000	28.89 ± 42.81	23.32 ± 45.12	0.51	0.610

### 3.5. The diagnostic agreement among the GLIM, PG-SGA, SGA, and PNI

We also evaluated the diagnostic agreement among the GLIM, PG-SGA, SGA, and PNI. When compared with the other three methods, the GLIM and PG-SGA were highly consistent in diagnosing malnutrition, with a kappa value of 0.814 (Table 6).

**Table 6:** Diagnostic concordances between the GLIM, PNI, PG-SGA, and SGA.

	**GLIM**		**Sensitivity**	**Specificity**	**Accuracy**	**Kappa**	***P***

	**Malnutrition**	**Normal**					
PG-SGA			0.811	0.989	0.910	0.814	<0.001
Malnutrition	120	28					
Normal	2	182					
SGA			0.737	0.981	0.954	0.752	<0.001
Malnutrition	14	5					
Normal	3	152					
PNI			0.406	0.860	0.630	0.265	<0.001
Malnutrition	104	152					
Normal	35	215					

### 3.6. The Univariate analysis of risk factors for nutritional risk and malnutrition

We analyzed the proportion of nutritional risk and malnutrition risk in different sociodemographic and disease treatment-related characteristics by the chi-square test (Tables 7, 8). The result showed that older age, lower BMI and tumorous were risk factors for nutritional risk and malnutrition.

**Table 7:** The Univariate analysis of risk factors for nutritional risk.

		**NRS2002 ≥3** **(*n* = 170)**	**NRS2002 <3** **(*n* = 336)**	***X* ^2^**	***P***
Sex	Male	114 (34.23%)	219 (65.77%)	0.177	0.693
	Female	56 (32.37%)	117 (67.63%)		
Age	≥65	91 (45.96%)	107 (54.04%)	22.285	0.000
	<65	79 (25.65%)	229 (74.35%)		
BMI (WGOC Categories^a^)	<18.5	51 (89.47%)	6 (10.53%)	97.986	0.000
	18.5–23.9	87 (31.52%)	189 (68.48%)		
	≥24	32 (18.50%)	141 (81.50%)		
Hypertension	Yes	45 (29.22%)	109 (70.78%)	1.900	0.184
	No	125 (35.51%)	227 (64.49%)		
Diabetes	Yes	25 (39.68%)	38 (60.32%)	1.195	0.318
	No	145 (32.73%)	298 (67.27%)		
Tumorous	Yes	147 (44.28%)	185(55.72%)	49.365	0.000
	No	23(13.22%)	151(86.78)		

^*a*^The Chinese Working Group on Obesity (WGOC) categories: a BMI <18.5 kg/m^2^ is considered underweight, 18.5 kg/m^2^ ≤ BMI <24.0 kg/m^2^ is considered normal weight, 24.0 kg/m^2^ ≤ BMI <28.0 kg/m^2^ is considered overweight and BMI ≥28.0 kg/m^2^ is considered obese (12).

**Table 8:** The Univariate analysis of risk factors for malnutrition.

		**Malnutrition** **(*n* = 139)**	**Normal** **(*n* = 367)**	***X* ^2^**	***P***
Sex	Male	96 (28.83%)	237 (71.17%)	0.902	0.401
	Female	43 (24.86%)	130 (75.14%)		
Age	≥65	74 (37.37%)	124 (62.63%)	16.012	0.000
	<65	65 (21.10%)	243 (78.90%)		
BMI (WGOC Categories^a^)	<18.5	49 (85.96%)	8 (14.04%)	120.317	0.000
	18.5–23.9	67 (24.45%)	207 (75.55%)		
	≥24	21 (12.14%)	152 (87.86%)		
Hypertension	Yes	38 (24.68%)	116 (75.32%)	0.868	0.387
	No	101 (28.69%)	251 (71.31%)		
Diabetes	Yes	21 (33.33%)	42 (66.67%)	1.217	0.292
	No	118 (26.70%)	325 (73.30%)		
Tumorous	Yes	122(36.75%)	210(63.25%)	41.700	0.000
	No	17(9.77%)	157(90.23%)		

^*a*^The Chinese Working Group on Obesity (WGOC) categories: a BMI <18.5 kg/m^2^ is considered underweight, 18.5 kg/m^2^ ≤ BMI <24.0 kg/m^2^ is considered normal weight, 24.0 kg/m^2^ ≤ BMI <28.0 kg/m^2^ is considered overweight and BMI ≥28.0 kg/m^2^ is considered obese (12).

## 4. Discussion

The prevalence of malnutrition among hospitalized patients undergoing hepatobiliary-pancreatic surgery is high (6), but the diagnosis has been controversial (21). In 2018, the American Society for Parenteral and Enteral Nutrition (ASPEN) and the Parenteral and Enteral Nutrition Society of Asia (PENSA) proposed the new GLIM criteria. These criteria provide new insights for the diagnosis of malnutrition. While, its varied prevalence in the same group may occur when diagnosed using different criteria or methods.

In this cross-sectional study, the GLIM criteria showed the lowest prevalence, and PNI defined the highest among the four methods. The prevalence was 36.75, 44.58, and 60.24%, as diagnosed by the GLIM, PG-SGA, and PNI, among 332 tumor patients. Among the 174 non-tumor patients, the prevalence was 9.77, 10.92, and 32.18% as determined by the GLIM, SGA, and PNI, respectively. A similar conclusion was reached by Miwa' study (22), the GLIM also showed the lowest prevalence (21%) among chronic liver disease patients when compared with SGA(35%), and RFH-GA (26%). As we know, the incidence of malnutrition varies relay on how estimator used the assessment tools, but regardless of the assessment tool, clinical outcomes in hospitalized patients with or without malnutrition are quite different.

The results of the Cohen's kappa statistic showed that the PG-SGA and GLIM had a relative high level of agreement (Kappa = 0.814, *P* < 0.001), the SGA and GLIM had a good level of agreement (Kappa = 0.752, *P* < 0.001), while the PNI and GLIM had fair level of agreement (Kappa = 0.265, *P* < 0.001). In the previous study, the PG-SGA and GLIM had a moderate level of agreement (kappa = 0.519, *P* < 0.001) in 360 esophageal cancer (EC) patients after esophagectomy (8). A possible explanation of the fair level of agreement between the GLIM and PNI may be that the PNI was a completely objective tool, including the level of serum albumin and the total number of peripheral leukocytes, while it did not consider BMI or reduced food intake. Meanwhile, the cutoff value of the PNI was different for different diseases. The widely used PNI cutoff value in gastrointestinal surgery is 45, and previous studies suggested that PNI ≤ 45 was significantly associated with poor prognosis in patients (23, 24). Moreover, Wang et al. (25) excluded patients with PNI ≤ 45, and the results showed that those with PNI≥ 55 had more difficulty obtaining a pathological complete response than patients with PNI >45 and <55. In Li Chen's study (26), the optimal cutoff value of PNI was 51. Therefore, the range of PNI cut-off values should be explored in clinical studies with large samples.

The possible reasons that the GLIM showed the lowest prevalence of malnutrition may be as follows: (1) in our study, most cases met the etiologic criteria because of the apparent reduced food intake, while did not meet the phenotypic one, mainly because that their weight loss did not meet the diagnostic criteria interval; (2) The GLIM criteria recommended the use of the dual-energy X-ray absorptiometry (DEXA), the computed tomography (CT) and the magnetic resonance imaging (MRI) to get the fat free mass index (FFMI), some other measurement of body composition such as bioelectrical impedance analysis (BIA) can be used as a reference. The normal values of the calculated muscle quality and the cut-point standards were still lack in China. So we excluded its measurement in our study, which may cause the lower prevalence of malnutrition diagnosed by the GLIM. In the present, some researchers have attempted to use the 15th percentile (p15) of the CC (A value <p15, male, 30 cm; female, 29 cm) as a positive for reduced muscle mass (8, 27). However, the optimal cut-off values for reduced muscle mass are debated. Other researchers discovered that using body composition measurements can further improve the GLIM criteria performance compared to using anthropometric measurements alone (28). There are racial and gender differences in normal values of the muscle mass and cut-off criteria, so the threshold of the related index of muscle mass reduction requires lager samples from different ethnic groups (e.g., Chinese, European) and higher quality research data.

Nutritional screening, nutritional assessment, nutritional intervention and monitoring were consisted of the standardized nutritional support therapy strategy. Among them, nutritional intervention plays a significant role (6). In our study, a low proportion of nutritional support was provided during hepatobiliary surgery. This may be because of the lack of recommendations for patients with nutritional risk by bedside physicians, and that of high-quality literature reports proving that nutritional support impacts the improvement in outcomes. So the medical staff should focus on the nutritional status and the nutritional intervention of hospitalized patients.

We found that the mean age of malnourished group was significantly higher than the well-nourished group according to all the four diagnostic tools. Moreover, other nutritional characteristics, including albumin and hemoglobin, were significantly lower in the malnourished group. These results are consistent with those of domestic and foreign studies (29, 30). Furthermore, the univariable analysis showed that older age, lower BMI and tumorous were risk factors for nutritional risk and malnutrition. The presence of benign or malignant disease due to the typical characteristics of malignancies may alter nutritional status (e.g., inflammation, central anorexia, metabolic disturbance worsen food intake, muscular status and energy reserve, which allow to define good nutritional status or malnutrition, are affected by tumorous).

Poulter (31) compared the GLIM, ESPEN, and ICD-10 criteria and identified 23.0%, 5.5%, and 12.6% of the cohort as malnourished, respectively. Xiaolin et al. compared the GLIM and PG-SGA criteria among cancer patients. They also found a lower prevalence of malnutrition diagnosed by the GLIM criteria (36.8 vs. 42.5%) (32). Regarding study strengths, this study is one of the first attempts to assess the diagnostic performance of four screening tools for malnutrition diagnosis, including the GLIM, PG-SGA, SGA, and PNI criteria, among hospitalized patients undergoing hepatobiliary-pancreatic surgery. Thus, our results show an important gap in this field.

However, our study has some limitations. First, it excluded reduction in muscle mass, which may have led to bias. Due to the short-staffed and the lack of equipment such as the dual-energy X-ray absorptiometry and bioelectrical impedance analysis in our study, we could not obtain the precise data about the muscle mass. Second, the GLIM calls for the use of the criteria in prospective and retrospective cohort studies to validate their application for clinical practice. However, our study design was retrospective and we used medical records, which inevitable had missing data, and the risk of selection bias was not negligible. Third, many studies observed the relationship between malnutrition and the adverse clinical outcomes of patients (33, 34). We mainly focused on short-term nutritional characteristics, and long-term clinical outcomes, such as complications, hospital readmission and mortality were not observed or reported. In the future, high-quality prospective clinical validation reports and prospective multi-center clinical application data analysis will be the basis for the improvement of the GLIM. The association between nutritional status and clinical outcome of patients assessed by various tools needs to be further explored.

Our study has practical implications. We assessed the prevalence of malnutrition, as defined by the GLIM, among hospitalized patients in hepatobiliary-pancreatic surgery and compared it with the other three criteria, which will help to reduce the bias of early clinical application of GLIM. Our findings provided some guidance and reference for the diagnosis of malnutrition, which may improve scientific and standardized nutrition management in hepatobiliary and pancreatic diseases. Meanwhile, we attempted to lay the foundation for further GLIM prospective clinical validation and health economics research. It is recommended to fill in the first page of the medical record with malnutrition information for disease codes E40-E46 and hopefully our findings can provide objective and valuable information for the diagnosis of malnutrition.

## 5. Conclusion

In conclusion, the incidence of nutritional risk and malnutrition was high among patients with hepatobiliary and pancreatic diseases, specifically in those with tumors. Among the four tools, the GLIM defines the lowest prevalence of malnutrition. The PG-SGA and GLIM had a relative high level of agreement. We should pay attention that there was only small part of patients received nutritional support in our current study. Further prospective and well-designed cohort studies were needed to validate the relevance of the GLIM criteria to explore the “gold standard” for clinical validity.

## Data availability statement

The original contributions presented in the study are included in the article/supplementary material, further inquiries can be directed to the corresponding author.

## Ethics statement

The studies involving human participants were reviewed and approved by the Ethics Committee of the Ningbo Medical Center Lihuili Hospital. The patients/participants provided their written informed consent to participate in this study.

## Author contributions

LZ contributed to conception, administration, writing, and editing of the manuscript. KJ, ZD, and RW contributed to nutritional risk screening and assessment of malnutrition. JF and LY contributed to data collection including disease diagnosis, age, sex, height, BMI, LOS, and laboratory data. All authors contributed to the article and approved the submitted version.
